# Valorisation diagnosis of waste from the decontamination of phosphogypsum leachates through a combined calcium carbonate/hydroxide process

**DOI:** 10.1016/j.heliyon.2024.e30610

**Published:** 2024-05-04

**Authors:** F.J. Soto-Cruz, S.M. Pérez-Moreno, E. Ceccotti, A. Barba-Lobo, J.P. Bolívar, M. Casas-Ruiz, M.J. Gázquez

**Affiliations:** a Department of Applied Physics, Marine Research Institute (INMAR), University of Cadiz, Campus de Excelencia Internacional del Mar (CEIMAR), Cádiz, Spain; b Research Centre of Natural Resources, Health and the Environment (RENSMA), University of Huelva, Campus de Excelencia Internacional del Mar (CEIMAR), Huelva, Spain

**Keywords:** Phosphogypsum leachate, Neutralisation, NORM waste, Valorisation diagnosis, Environmental impact

## Abstract

Phosphogypsum is an industrial waste considered as naturally occurring radioactive material. Stack disposal and exposure to the environmental condition involve the production of acid leachates with high potential pollutant loads as heavy metals and radionuclides. In this study, a sequential neutralisation process was applied for cleaning the generated releases, and the two obtained residues were characterised from the physical-chemical and radiological point of view before their valorisation. The cleaning process was made up of two steps: the first one using calcium carbonate until pH = 3.5, and the second one using calcium hydroxide until pH = 12. The residue obtained in the first step was mostly calcium fluoride, while in the second step most phosphates were precipitated, mainly as hydroxyapatite. The final liquid was treated to reduce pH lower than 9, which is the limit included in the current directive for discharges of liquid effluents into coastal waters. The main conclusion was that the solids from the first step could be valorised as an additive in the manufacture of commercial Portland cements and ceramics, while the solids from the second step could be used as raw material for the phosphoric acid manufacture.

## 1 Introduction

Phosphogypsum (PG) is the main waste generated during the wet process manufacture of phosphoric acid, wherein rock phosphate and sulfuric acid are used as raw materials [1]. The phosphoric acid is crucial to meet the high global demand for fertilisers, driven by the vast crop production of the agricultural sector [2]. The global supply of phosphoric acid would increase due to the continuous rise in global population [3,4]. It is estimated that 3000 Mt of PG is stocked in at least 65 countries all over the world (Spain, Morocco, China, and Tunisia, among others) [5,6], and PG is annually generated in a ratio of 175 Mt/y [7]. However, only 15% of PG produced worldwide is recycled [8].

In Huelva there was a fertiliser complex working from 1965 to 2010 that generated 120 Mt of PG, and around 85% was stored in big piles in the right bank of the Tinto estuary (Fig. S1). Specifically, in the Huelva province, the used ores were mainly fluorapatite, and the wet process is given by the following equation:

(1) $${Ca}_{5}{\left({PO}_{4}\right)}_{3}F+5H_{2}{SO}_{4}+10H_{2}O\;\to \;3H_{3}{PO}_{4}+5{CaSO}_{4}2H_{2}O+HF$$

As can be seen in Eq. (1), hydrogen fluoride gas is also generated as a non-main product collected and sent to scrubbers to be absorbed in water, so a hydrofluoric acid weak effluent is produced. This effluent flow is mixed with the PG and deposited onto the stockpiles [9].This PG repository is divided into 4 zones (Fig. S1 in the Supplementary Material). They are openly exposed to environmental weathering, thus contributing to the formation of leachates from raining periods. The piles have a series of perimeter channels to collect leachates from PG weathering, but there are many points and diffuse sources of edge outflows that discharge into the estuary [10].

The phosphate industry is classified as a naturally occurring radioactive material (NORM) activity, since raw material often contains natural radionuclides (RNs) with activity concentrations greater than the thresholds set by both the EU Directives [11] and the Spanish Nuclear Safety Council (CSN in Spanish).

The phosphate rock used in Huelva from Morocco contains activity concentrations of ^238^U and daughters around 1.5·10^3^ Bq/kg, which is about 50 times greater than those found for typical soils [12], while the RN concentrations belonging to the ^232^Th series and ^40^K were around 10 Bq/kg and 30 Bq/kg, respectively [13], which are lower than those found for typical soils. Most uranium in raw material (more than 85% of U) remains in dissolution together with the phosphoric acid, while more than 95% of the reactive elements (^226^Ra, ^210^Pb, and ^210^Po) and 70% of ^230^Th remain in the PG [[13], [14], [15]].

The main problem addressed in this work was reducing the environmental emission of RNs and heavy metals (HMs) from the Huelva unrestored PG piles (Zones 2 and 3, in Fig. S1 in the Supplementary Material)), which can be released by lateral outflows until reaching the estuary. Previous studies have characterised the leachates from the PG stacks and collected in the perimeter channel, thus showing that these waters have high acidity (pH < 2) and high concentrations of total phosphorus (10^4^ mg/L), fluorides (5·10^3^ mg/L), sulphates (4·10^3^ mg/L), U isotopes (25 mg/L), and metals such as K (250 mg/L), Mg (600 mg/L), Ca (1600 mg/L), Na (3.5·10^3^ mg/L), Cl (2·10^3^ mg/L), and Br (100 mg/L) [7,16].

Neutralisation is the most common process used for acid effluents treatment as the precipitation of most metals can be produced by increasing the pH up to alkaline conditions. Few studies focused on the treatment of the phosphogypsum leachates (PGL) have been developed [[17], [18], [19]]. In fact, the fertilizer company initially proposed a cleaning process in four sequential steps. In the first one, calcium carbonate is added up to pH = 3.6, and then calcium hydroxide is used up to pH = 10.5. During this last step, the ammonium vapors are removed with water. Finally, the pH is reduced to 7–8 by using sulfuric acid to verify the liquid discharges regulation [20].

Pérez-Moreno et al. [21] developed and optimised the cleaning process of these acidic leachates by using different alkaline chemical reagents such as Ca(OH)_2_, CaCO_3_, NaOH, Na_2_CO_3_, Mg(OH)_2_, and MgCO_3_ on a laboratory scale. The most appropriate reagents for cleaning pollutants were Ca(OH)_2_ and CaCO_3_, being this process divided into two steps, in the first one (pH around 3–4) fluorite is mainly formed, and hydroxyapatite (with pH = 12) is obtained in the second step [22]. These products have a high potential recovery, and their valorisation is a feasible process. In this work, the neutralisation process of the PGL was carried out with Ca(OH)_2_ as it greatly removes contaminants [21]. In addition, CaCO_3_ was also selected to compare both alkaline reagents only in the first step because CaCO_3_ only reaches a pH of 6 [21].

This work aims to comprehensively characterise the residues obtained by using Ca(OH)_2_ and CaCO_3_, and subsequently to perform a valorisation diagnosis based on the results.

## 2 Materials and methods

### 2.1 Sampling

A leachate sample was taken in the perimeter channel from zone 2 of the PG stacks (Fig. S1), which collects all the leachate produced in this zone above its level. The sample (around 20 L) was collected in September 2020. The physicochemical parameters (i.e., pH, electrical conductivity (EC), and oxidation reduction potential (ORP)) were measured in situ by using a portable equipment (Crison MM40+) equipped with a multiparametric probe. Afterwards, the sample was filtered through a vacuum system in the laboratory by using cellulose nitrate filters of 0.45 μm pore size.

### 2.2 Design of experiments

**Fig. 1:**
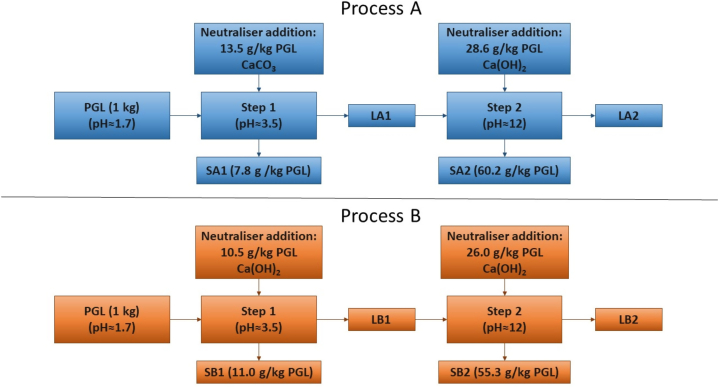
Diagram of the sequential neutralisation processes A and B. The masses of the reactants and the resulting solids are included.

The design of experiments was based on two processes conducted in parallel: Process A was conducted using calcite (CaCO_3_) in the first step until pH = 3.5, and then hydrated lime (Ca(OH)_2_) in the second step until pH = 12, while Process B was conducted with hydrated lime in both steps (Fig. 1). These reagents were selected to evaluate the various solids formed in each case.

In both processes, 2 L of phosphogypsum leachate were used, and the experiments were made by duplicate, consisting of adding the specific alkaline reactive whose mass was previously determined considering the pH that needs to be reached.

The experiment consisted of adding the alkaline agent in quantities of 2–5 g, stirring the solution with a magnetic system. The pH was measured in intervals of 5–10 min, when it was stable, and the desirable pH was reached (Fig. 1). Afterwards, the solution was filtered using a cellulose nitrate filter of 0.8 μm pore size, obtaining both the liquid fraction and the solid fraction, which was dried in oven at 50 °C until constant mass. The solids were weighed and reserved for posterior analysis, and the parameters such as pH, EC, and ORP were measured in the liquid fraction.

The obtained samples were codified considering the Process (A or B), the step of the process (1 or 2), and the fraction (liquid or solid), so the sample LA1 corresponded to the liquid fraction of Step 1 from Process A. In short, 4 sampling batches were obtained for each process, 2 for each step (liquid and solid).

### 2.3 Characterisation techniques

#### 2.3.1 Multielemental analysis

Trace elements were measured by ICP-MS/OES at the Activation Laboratories (ACTLABS, Ontario (Canada)) using a spectrometer PerkinElmer Sciex ELAN 9000 and Agilent Axial 730-ES respectively.

Major elements were determined by X-ray fluorescence (model ZETIUM) at the Centre for Research, Technology, and Innovation of the University of Seville (CITIUS). The OMNIAN semi-quantitative method is a wavelength dispersive X-ray fluorescence (WDXRF). At the SGI X-Ray Laboratory, the relative uncertainties for each element present in the sample were obtained according to [23].

Regarding the ion composition, samples were analysed by the ion chromatographer DIONEX DX-120, which is a complete system with an isocratic bomb, a conductivity detector, an automatic sampler AS40.

The quality control of all analytical data obtained by the four previous characterization techniques was carried out by using a blank reagent, a certified reference material (CRM), and replicas of samples, thus obtaining accuracies of the analytical data ranged from 5% to 10%.

#### 2.3.2 Mineralogical analysis

The XRD patterns were obtained using a Bragg-Brentano geometry powder X-ray diffractometer with a Cu tube (Bruker, model D8 Advance A25). A semi-quantitative method was used with the following conditions: Δ2θ = 3–70°; step = 0.015°; t = 0.1 s; tube conditions: 40 kV and 30 mA; divergence slit: fixed 0.5°; sample rotated at 30 rpm, and nickel filter. The Diffrac Eva Phase Identification (DIFFRAC.EVA) software was used to identify the crystalline phases, using the PDF-4 database (ICDD). The quantification was carried out using the Rietveld method (the DIFFRAC.TOPAS software), for which zincite (ZnO) was selected as an internal standard and at a proportion of approximately 15% by weight to determine the amorphous fraction.

#### 2.3.3 Radioactive analysis

With respect to the measurements of alpha emitters, alpha-particle spectrometry was applied, using an EG&ORTEC system with an integrated Octete PC PLUS. Likewise, the Maestro software was used for data analysis and acquisition. The samples were firstly subjected to the sequential separation method by using tributyl phosphate (TBP) to absorb actinides [24]. Afterwards, Po was self-deposited onto silver discs, and U isotopes were electrodeposited onto stainless steel discs.

Regarding the measurements of gamma emitters, an extended range (XtRa) high-purity germanium detector (model GX3519, Canberra) was used as it covers a very wide energy range (from 0 to 3 MeV). Within this range, all the gamma emitters of interest are present. The relative efficiency was 38.4% at 1332 keV (^60^Co) in relation to a 3″ × 3″ NaI (Tl) detector, a full width at half maximum (FWHM) of 1.74 keV and 0.88 keV at 1332 keV (^60^Co) and 122 keV (^57^Co), respectively, and a peak-to-Compton ratio of 67.5:1.

For both spectrometric techniques, blanks, replicas and CRMs were used to make a quality control of the results [25,26].

### 2.4 Leaching test

This test was conducted according to the UNE–EN–12457-4 standard [27]. It is an appropriate test to evaluate the mobility of both organic and inorganic compounds in solids wastes.

After conducting the leaching test, the leached concentration (C_i_) of a chemical species “x” (mg/kg of waste) was calculated with the following equation:

(2) $$C_{i}=C\;.\;\left[\frac{L}{M_{D}}+\frac{MC}{100}\right]$$

Where C_i_ is the released concentration of the pollutant in the solid phase at L/S = 10 (in mg/kg dry mass); C is the content of a particular constituent in the eluate (in mg/L); L is the volume of the leaching used (in L); MC is the wet ratio content expressed as a percentage of dry mass, and $M_{D}$ is the dry mass of the test portion (in kg).

Additionally, the transfer factor (TF) was calculated as follows:

(3) $$TF\;\left(\%\right)=\frac{C_{i}\;\left(\frac{mg}{kg}\right)}{C_{i,s}\;\left(\frac{mg}{kg}\right)}\;x\;100$$

Where $C_{i}$ is the concentration of the element “i” in the leaching test at L/S = 10 (in mg/kg dry mass), and $C_{i,s}$ is the concentration of the element “i” in the solid.

### 2.5 Precipitation efficiencies

The material balances were done to determine the proportion of the contaminants removed in all cases. The percentage of precipitation of an element “x” during the process or precipitation efficiency (PE) was defined as the relationship between the amount of an element in the resulting solid and the amount of that element in the initial solution. Thus, PE of a certain element (x) was determined according to the following equation:

(4) $$PE\;\left(\%\right)=\left(1-\;\frac{C_{1,2}.\;V_{1,2}}{C_{i}.\;V_{i}}\right).100$$

Where Ci (mg/L) is the concentration of the element in the initial solution “i”, C1; 2 (mg/L) is the concentration of the element in the final solution “f” (Step 1 or Step 2), V1; 2 (L) is the final volume of the effluent, and Vi (L) is the volume of the initial solution.

As for Ca, the added amounts of CaCO_3_ or Ca(OH)_2_ were considered. PEs for Ca were therefore calculated with the following equation:

(5) $$PE\;\left(\%\right)=\left(1-\;\frac{C_{1,2}.\;V_{1,2}}{C_{i}.\;V_{i}+m_{i\;added}}\right).100$$

Where m_i added_ is the amount (in mg) of added Ca (Fig. 1).

## 3 Results and discussion

### 3.1 Physicochemical characterisation of PGL

PGL properties changes throughout the year due to the seasonal behaviour of the climate in Huelva, with extreme values between wet and dry season [28,29]. This study includes the physicochemical and radiological characterisation of PGL from a dry season sample (Table S1 in the Supplementary Material), as well as the composition of the background value corresponding to seawater (SW) [30]. The PGL presented a pH of 1.7, thus meaning an extremely acid solution compared with SW (potential receptor medium), which had a neutral/alkaline (pH ≈ 7.8). Moreover, the PGL had a half electrical conductivity (EC = 30.2 mS/cm) and higher redox potential (Eh = 656 mV) in relation to the background (seawater of the estuary), which had 61.0 mS/cm and 460 mV, respectively.

Several elements had relatively high concentrations, such as As, Cr, Cd, Pb, and Cu, which exceeded in 3 orders of magnitude the concentrations in SW. The concentrations of Fe, Mn, U, Ni, and Zn were 2 orders of magnitude higher, and that of Al was 1 order of magnitude higher (Table S1 in the Supplementary Material).

In relation to the concentrations of anions (Table S1 in the Supplementary Material), phosphates and fluorides levels were 40 times and 20 times greater than those contained in SW, respectively. These concentrations were similar to those found in other studies performed in the same zone [7,21,31], as well as to the PGL from other places [18,32].

The activity concentrations of natural RNs were also included in Table S1. The PGL sample mainly contained about 300 Bq/kg of ^238,234^U, 40 Bq/kg of ^210^Po, 230 Bq/kg of ^234^Th, and 60 Bq/kg of ^210^Pb, where this high concentration was remarkable as the background values 4-5 orders of magnitude were exceeded.

According to the results previously obtained from the physicochemical and radioactive characterisations of PGL, leachate waters should be collected and treated before discharging them into the estuary.

### 3.2 Evolution of contaminants during the sequential neutralisation steps

The physicochemical and radioactive characterisations of liquid and solid fractions from the two steps are shown in the following sections.

The elemental composition of the liquid and solid fraction obtained from the sequential neutralisation process is included in Tables S2 and S3 in the Supplementary Material, as well as their physicochemical data and the original leachate data used in this treatment process.

Various mass amounts of precipitated solid were obtained for each step and process during neutralisation. Fig. 1 includes the mass of formed solid and the alkaline reactive mass per kilogram of leachate to calculate the material balances.

#### 3.2.1 Step 1

##### 3.2.1.1 Liquid samples

The pH, electrical conductivity (EC), and redox potential (Eh) were determined. The pH of this step was 3.5, and the EC of both processes had similar values (24.3 mS/cm and 23.8 mS/cm for LA1 and LB1, respectively), thus reducing the electrical conductivity compared to the original PGL of around 20%, as expected due to the reduction of dissolved salts. The Eh was 557 mV and 553 mV for LA1 and LB1, respectively, around 16% lower than the PGL.

**Fig. 2:**
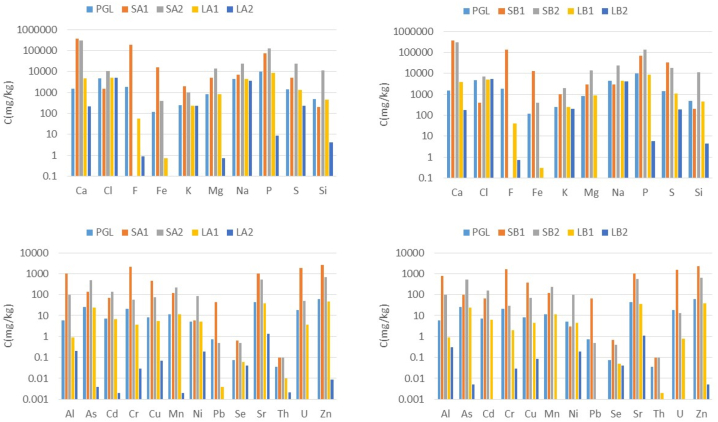
Major and minor element concentrations (mg/kg) of the liquids (LA1, LA2, LB1, and LB2) and solids (SA1, SA2, SB1, and SB2) generated in both decontamination processes (A and B) compared with PGL.

Regarding the elemental composition (Fig. 2), in Process A the concentration of Al, Cr, F, Fe, Pb, Th, and U were reduced more than 75% in the first step of the neutralisation process (LA1), while the results were similar in Process B, where Ca(OH)_2_ was used.

**Fig. 3:**
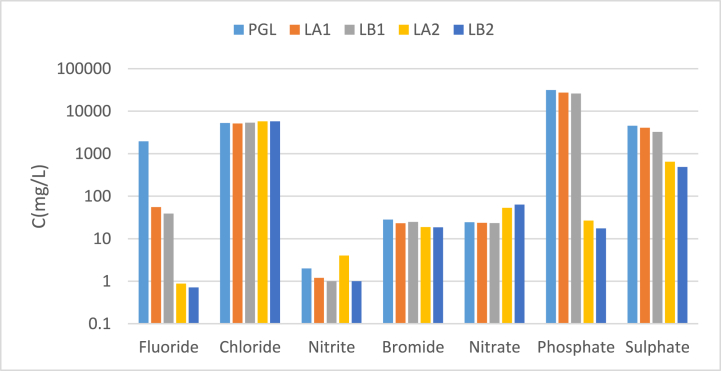
Anion concentration (mg/L) of PGL, and the liquid fraction obtained in the decontamination process (LA1, LA2, LB1, and LB2).

The concentrations of phosphates and sulphates did not significantly decrease at pH = 3.5 in Step 1 of both processes (LA1 and LB1) (Fig. 3), while the concentrations of fluorides were reduced around 95%.

The behaviour of natural RNs, especially ^210^Po, ^238^U and ^210^Pb, during the Step 1 for both processes (A and B) is included in Table 1. All concentrations of the natural RNs decreased in both processes, with Ca(OH)_2_ having better removal efficiency for these RNs.

**Table 1:** Radioactive characterisation of Step 1 for Process A and B by alpha-particle spectrometry, gamma-ray spectrometry, and ICP-MS in (Bq/kg).

	Isotope	PGL	Process A		Process B	
			LA1	SA1	LB1	SB1
		Activity concentration (Bq/kg)				
**Alpha**	^**210**^**Po**	39.0 ± 0.2	6.6 ± 0.2	5120 ± 80	0.20 ± 0.04	4370 ± 140
	^**238**^**U**	248 ± 12	39.6 ± 1.2	25900 ± 400	10.1 ± 0.3	20600 ± 600
**ICP**	^**232**^**Th**	0.04 ± 0.06	< 0.002	< 0.4	< 0.002	< 0.4
**Gamma**	^**210**^**Pb**	60 ± 3	< 1	8500 ± 500	< 1	6100 ± 300
	^**226**^**Ra**	< 2	< 2	39 ± 3	< 2	14 ± 2
	^**228**^**Ra**	3 ± 1	< 1	< 20	< 1	< 20
	^**228**^**Th**	< 1	< 1	< 5	< 1	3 ± 2

##### 3.2.1.2 Solid samples

**Fig. 4:**
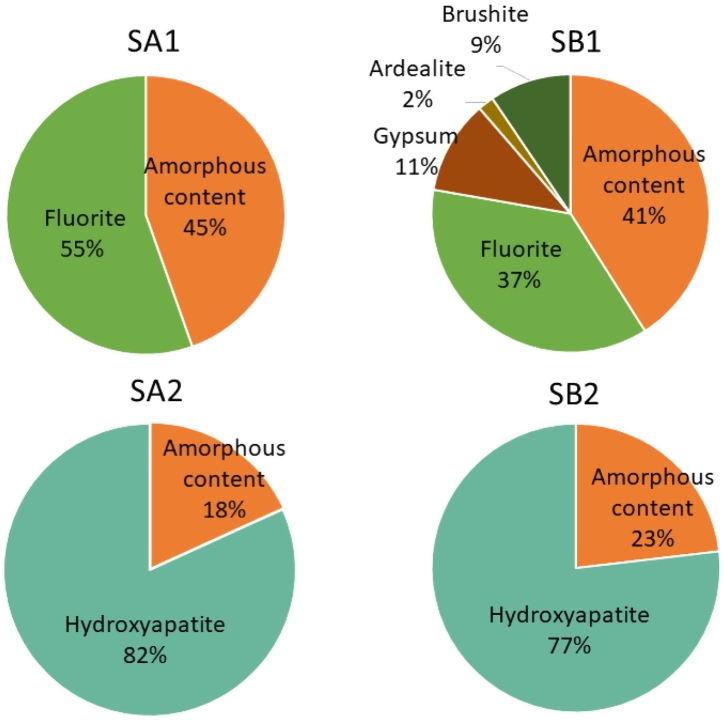
Mineralogical analysis by XRD of solids phases generated in the decontamination process (SA1, SA2, SB1, SB2).

The elemental composition of the two precipitated solids at pH = 3.5 by using CaCO_3_ (SA1) and Ca(OH)_2_ (SB1) for the neutralisation is shown in Fig. 2. In addition, the composition of the PGL was also included for comparative purposes. Both solids (SA1; SB1) contained Ca (36.5% and 35.7%, respectively) and F (18.8% and 13.6%, respectively) whose concentrations were 2 orders of magnitude greater than those found for the PGL. Additionally, P with a concentration close to 0.9% was 1 order of magnitude greater than in the PGL. Fig. 4 shows 55.4% of fluorite content (CaF_2_) and 44.6% of amorphous content for SA1, while in SB1, fluorite content was lower (36.8%), and 10.9% of gypsum (CaSO_4_·2H_2_O), 9.5% of brushite (CaHPO_4_·2H_2_O), and 1.9% of ardealite (Ca_2_(SO_4_)(HPO_4_)·4H_2_O) were also formed, with 41.0% of amorphous content. These mineral phases were compatible with the XRD pattern (Fig. S2 in the Supplementary Material).

Other elements, such as S, Cd, Cu, As, Mn, Pb, Sr, and Zn, showed concentrations of more than 1 order of magnitude greater than the PGL, while the concentrations of Fe, Al, Cr, and U were 2 order of magnitude greater compared with the PGL (Fig. 2). Additionally, elements such as P, S, Zn, Cd, As, Cr, and U were 1–2 order of magnitude greater than unperturbed soils [33].

There was no presence of Fe, Al, Na, and Mg in the compounds obtained by XRD in both processes, so these elements were in the amorphous fraction.

The activity concentration of ^238^U, ^210^Po, and ^210^Pb (Table 1) were approximately 3 orders of magnitude greater than typical soils [12], and significantly greater compared to the levels of NORM wastes 1 Bq/g [34]. The reason for these high concentrations was the small mass obtained in solid 1 (Fig. 1). Except for ^232,228^Th and ^226^Ra, that were below the limit of detection (LD), in agreement with the low concentrations in the PGL, the greatest activity concentrations of all RNs were found in the solid from Step 1 (SA1 and SB1). The ^234^U/^238^U activity ratio was very close to one, which meant that the isotopes were in secular equilibrium, expectable for a liquid obtained by the dissolution of a mineral of several millions of years.

#### 3.2.2 Step 2

##### 3.2.2.1 Liquid samples

The final pH of the second step was 12. Furthermore, the EC of both processes had similar values (19.1 mS/cm and 18.7 mS/cm for LA2 and LB2, respectively), thus dropping the EC in comparison with the original PGL as expected because of the reduction in the dissolved salts. In addition, Eh changed from 656 mV (a positive value in the PGL) to 163 mV and to 177 mV for LA2 and LB2, respectively.

Most elements were reduced in a range of 1–4 orders of magnitude. Some of them, such as Pb, Cd, Mn, and U, reached concentrations lower than the LD of the technique (ICP-MS), as Fig. 2 shows.

Elements such as Cl, K, and Na had similar concentrations for the original PGL and for the second step of both processes (A and B). These elements were therefore conservative elements and were not affected by the process.

There was no variation in the composition of nitrates, chlorides, and bromides obtained in the two processes (Fig. 3). This fact was expectable as these anions did not form insoluble sales with Ca [10]. The fluorides in the liquid fraction were in a very low proportion, i.e., 0.9 mg/L (LA2) and 0.7 mg/L (LB2), respectively. Furthermore, most of phosphates and sulphates precipitated in this step. When Ca(OH)2 was used, the concentrations of phosphates and sulphates for the liquid fraction at Step 2 were 1.3 and 1.5 times lower, respectively, compared to the use of CaCO_3_. This was an important difference between the two liquid discharge processes.

The concentrations of all natural RNs decreased in both processes in the final liquid (LA2 and LB2), thus being close to the LD (Table 2).

**Table 2:** Radioactive characterization of Step 2 for processes A and B by alpha-particle spectrometry, gamma-ray spectrometry and ICP-MS in (Bq/kg).

	Isotope	PGL	Process A		Process B	
			LA2	SA2	LB2	SB2
		Activity concentration (Bq/kg)				
**Alpha**	^**210**^**Po**	39.0 ± 0.2	0.01 ± 0.02	58 ± 5	0.01 ± 0.03	16 ± 1
	^**238**^**U**	248 ± 12	0.02 ± 0.01	750 ± 20	0.10 ± 0.02	210 ± 10
**ICP**	^**232**^**Th**	0.04 ± 0.06	< 0.002	< 0.4	< 0.001	< 0.4
**Gamma**	^**210**^**Pb**	60 ± 3	< 1	< 100	< 1	< 100
	^**226**^**Ra**	< 2	< 2	< 20	< 2	< 20
	^**228**^**Ra**	3 ± 1	< 1	< 40	< 1	< 50
	^**228**^**Th**	< 1	< 1	< 15	< 1	< 20

##### 3.2.2.2 Solid samples

As for solids from the second step, the content of Ca and P was 30.5% and 12.4%, respectively, for Process A (SA2), and 30.5% and 13.6%, respectively, for Process B (SB2). These values were expected because the content of calcium phosphates precipitated from the PGL was high due to the increase of pH by adding calcium in form of Ca(OH)_2_ during neutralisation. The mineral compounds of solids therefore corresponded to hydroxyapatite (HA), which was a compound of calcium phosphate (Ca_5_(PO_4_)_3_OH) with a concentration of 81.8%, with 18.2% of amorphous content in Process A. In Process B, these percentages were slightly lower (76.9% of HA, and 23.2% of amorphous content). This fact should be considered in order to generate the most quantity of waste with high purity for its posterior valorisation. Theses mineral phases were compatible with the XRD pattern (Fig. S3 in the Supplementary Material).

After calculating the concentration of Ca associated to the crystalline phase in relation with the total concentration of Ca, there was 15.3% and 13.3% of Ca amorphous in solid SA2 and SB2, respectively. Additionally, part of the content of P was also amorphous, 8.7% in Process A and 10.6% in Process B.

The concentrations of elements such as F and Fe were close to their LD in Step 2. Elements such as S, Mg, Na, Cd, Mn, As, and P had concentrations in a range of 1–4 times greater than solid of Step 1 in both processes. The concentration of Ni was 1 order of magnitude higher, while for Al, Cr, Zn, Cu and U, the concentrations were in a range of 1–2 orders of magnitude lower than those obtained in Step 1.

The concentrations of ^238^U, ^210^Po and ^210^Pb decrease (∼ 95–100%) in the second step, when the pH increased to 12 (Table 2). Nevertheless, the activity concentration of ^238^U in both solids was approximately 20 times higher than the worldwide median value for natural soils (35 Bq/kg) [12]. Notably, the LD of ^228^Th, ^210^Pb and ^226, 228^Ra in SA2 and SB2 were relatively high because a very small amount of mass was measured by gamma-ray spectrometry.

#### 3.2.3 Precipitation efficiencies

**Fig. 5:**
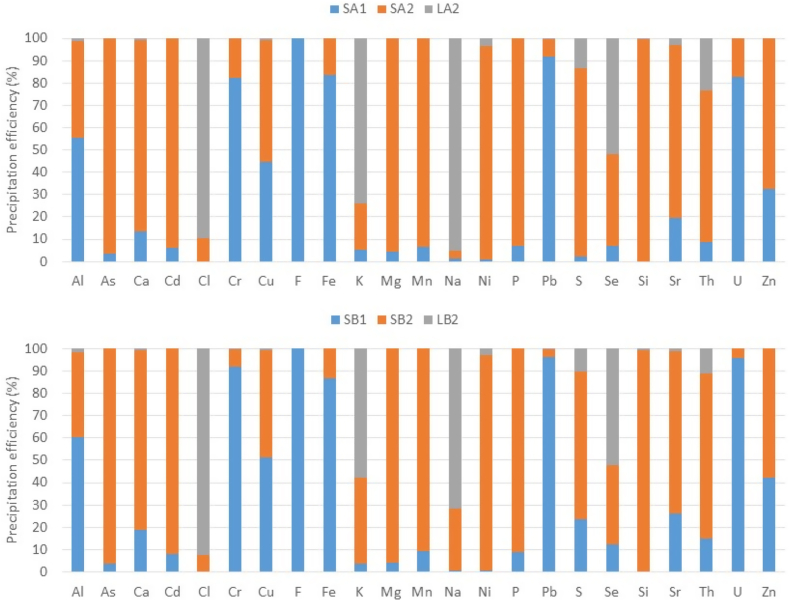
Precipitation efficiencies (%) of the element present in solids (SA1, SB1, SA2, SB2) and final effluent (LA2, LB2) obtained in the decontamination process.

Fig. 5 shows the balances of the complete processes. The total precipitation efficiencies for both processes were similar. As a main conclusion, from the 100% of the PGL in the process, some elements such as Cr, F, Fe, Pb, and U were transferred (from 80% to 100%) to solids in Step 1, while for other elements such as As, Ca Cd, Mg, Mn, Ni, P, S, Si, Sr, and Th, a percentage lower than 30% was transferred to solids in Step 1. In the case of Step 2, more than 66% of elements such as As, Cd, Mg, Mn, Ni, P, S, Si, Sr, and Th were transferred to solids.

Finally, some elements such as Cl, K, Na, and Se were not affected by the sequential neutralisation process, remaining in the final liquid with a percentage of transference greater than 50%.

Regarding the transfer percentage in the liquid final effluent for the rest of the elements, it was negligible (lower than 1–1.5%) for elements such as Al, As, Ca, Cd, Cr, Cu, F, Fe, Mg, Mn, P, Pb, Si, U, and Zn, occurring only transference of Ni, S, Sr, and Th, thus obtaining a TF greater than 23%.

The exact values of precipitation percentages and the concentration of each element in the steps of both processes are shown in Table S4 in the Supplementary Material. The relative uncertainties of the material balances were estimated for all elements around 10–15%, excepting for Al, Fe, Cd, Cl, Mn, Pb, Th, and Si, for which uncertainties were greater. The reason could be due to the trace elements that contain the alkaline reagents, especially in the case of Ca(OH)_2_. These data can be found in Table S5 in the Supplementary Material**.**

### 3.3 Environmental implications

#### 3.3.1 Liquid discharge limits

According to the criteria of Directive 2000/60/EC of the European Parliament and of the Council of October 23, 2000 in Andalusia, the discharges of the liquid effluents into coastal waters are controlled by Decree 109/2015, which approves the regulation of discharges to the Hydraulic Public Domain and the Maritime-Terrestrial Public Domain of Andalusia, in which the emission limit value is established (Table 3).

**Table 3:** Final effluent obtained and its comparison with limit values for discharge in coastal waters (Decree 109/2015) And World Human Organization (WHO) and Royal Decree 1620/2007 for agricultural use of water. N.M.: Not measured.

Element	Process		Limit value Decree 109/2015 (mg/L)	WHO (2006)	Royal Decree 1620/2007
	LA2	LB2			
	Concentration (mg/L)				
**Al**	0.2	0.3	10	5.0	–
**As**	0.004	0.005	1.2	0.1	0.1
**Be**	< 0.02	< 0.02	–	0.1	0.1
**B**	N.M.	N.M.	3	0.1	–
**Ca**	213	180	–	–	–
**Cd**	0.002	< 0.001	0.014	0.01	0.01
**Cr**	0.03	0.03	0.36	0.1	0.1
**Co**	< 0.001	< 0.001	–	0.05	0.05
**Cu**	0.07	0.09	0.9	0.2	0.2
**Fe**	< 0.1	< 0.1	3.6	5	–
**K**	227	204	–	–	–
**Mo**	0.01	0.003	–	0.01	0.01
**Mg**	0.7	0.1	–	–	–
**Mn**	0.002	0.001	9	0.2	0.2
**Na**	4030	4020	–	–	–
**Ni**	0.19	0.19	0.7	0.2	0.2
**P**	8.7	5.7	60	–	0.5
**Pb**	< 0.001	< 0.001	0.26	5	–
**S**	236	184	–	–	–
**Se**	0.04	0.04	0.2	0.02	0.02
**Si**	4.1	4.3	–	–	–
**Sn**	< 0.001	< 0.001	20	–	–
**Ti**	< 0.01	< 0.01	5	–	–
**U**	< 0.001	< 0.001	–	–	–
**V**	0.002	0.001	–	0.1	0.1
**Zn**	0.009	0.005	1.8	2	–
**Anions**					
**F**^−^	0.9	0.7	17	–	–
**Cl**^−^	5770	5750	–	–	–
**Br**^−^	18.7	18.6	–	–	–
**NO**_3_^−^	53.3	63.4	110	–	10
**PO**_4_^−^	26.7	17.5	165	–	–
**SO**_4_^−^	645	488	–	–	–
**pH**	12	12	9.5–5.5	–	–
**Conductivity (mS/cm)**	18.9	18.7	–	–	3

The final effluent obtained in the sequential neutralisation process of both treatments are also included in Table 3**.** The composition of the effluent using CaCO_3_ and Ca(OH)_2_ met all the requirements for discharge concentrations. In addition, the concentrations of fluorides, nitrates. and phosphates were also under the limit. The only exception was the requirement for the pH because the pH reached in the final step was up to 12.

A treatment should therefore be studied to decrease the effluent pH and to discharge these liquid effluents into coastal waters. A qualitative experiment was conducted by bubbling the final liquid with air, and the pH decreased from 12 to 8–9, so it could be discharged into coastal waters. Thus generating a solid fraction (Fig. S4 in the Supplementary Material) whose diffractograms corresponded with CaCO_3_ (Fig. S5 in the Supplementary Material). This reaction occurs following Eq. (6):

(6) $${CO}_{2}\left(aq\right)+2{OH}^{-}+{Ca}^{2+}\to \;CaCO_{3}\left(s\right)+H_{2}O$$

#### 3.3.2 Landfill disposal admission

The European Commission regulated waste disposal in landfill by Directive 1999/31/EC, and the waste acceptance criteria were established for each class of landfill in Royal Decree 646/2020. The leaching test was therefore conducted following the UNE-EN_12457-4 standard.

Table 4 shows that SA1 and SB1 were considered hazardous wastes as the concentrations of As, Cd, and Zn exceeded the concentration included in the Royal Decree. For solids in the second step, SA2 would be classified as hazardous waste because As also exceeded the limits of 2 mg/kg. However, SB2 had a 75% greater concentration, thus disposing in the landfill as it exceeded the 25 ppm established by the regulations. In the case of RNs, there is no regulation for landfill disposal. However, the TF was less than 0.1% for ^238^U for all solids, and less than 0.1% for ^210^Po for solids from Process A (SA1, SA2). In solids from Process B, the TF was less than 10% for ^210^Po.

**Table 4:** Concentration (mg/kg), activity concentration (Bq/L) and transfer factor (%) (in parentheses) obtained after leaching test of solids from both steps and both processes and its comparison with Royal Decree 646/2020 (Landfill disposal Regulations).

Element	Process A		Process B		Royal Decree 646/2020		
	SA1	SA2	SB1	SB2	Inert Waste	No Hazardous waste	Hazardous waste
	Concentration (mg/kg)						
**Al**	4.0 (0.4)	1.0 (0.4)	6.8 (1.0)	1.0 (0.5)			
**As**	22 (17)	8.2 (1.7)	19 (20)	**33** (6.3)	0.5	2	25
**Ca**	4200 (1.6)	90 (0.03)	9300 (3.6)	31 (0.01)			
**Cd**	2.2 (3.0)	1.0 (0.7)	1.6 (2.4)	1.0 (0.7)	0.04	1	5
**Cr**	0.22 (0.01)	0.31 (0.6)	0.05 (0.003)	0.1 (0.4)	0.5	10	70
**Cu**	2.3 (0.6)	0.074 (0.1)	1.3 (0.4)	0.048 (0.02)	2	50	100
**K**	710 (30)	870 (71)	220 (18)	910 (59)			
**Mg**	570 (9.8)	470 (3.1)	530 (18)	330 (1.9)			
**Mn**	8.7 (7.4)	1.0 (0.4)	8.9 (7.5)	1.0 (0.4)			
**Na**	2700 (43)	12000 (61)	1300 (49)	8600 (45)			
**Ni**	2.8 (48)	0.31 (0.3)	2.2 (85)	0.31 (0.4)	0.4	10	40
**P**	7200 (9.9)	78 (0.1)	7200 (11)	540 (0.4)			
**S**	1600 (32)	5200 (23)	4900 (16)	4600 (26)			
**Sr**	23 (2.8)	0.29 (0.1)	37(5.7)	0.10 (0.02)			
**Zn**	57 (2.3)	0.011 (0.002)	26 (1.1)	0.02 (0.003)	4	50	200
**Isotopes**	**Activity concentration (Bq/L)**						
^**210**^**Po**	0.32 (0.1)	0.24 (0.1)	0.17 (2.9)	0.16 (10)			
^**238**^**U**	0.30 (0.0)	< 0.004 (0.0)	0.10 (0.1)	< 0.001 (0.0)			

The TFs are included in Table 4, showing that elements such as As, K, Mg, Na, Ni, P, and S were more mobile in Step 1 of Process A and B, with K and S being more mobile in Process A (with CaCO_3_), and Ni in Process B (with Ca(OH)_2_).

However, in the final step of the processes, only elements such as K and Na had a high TF, which were the conservative elements not affected by the neutralisation sequential process. The rest of the elements had a TF lower than 5% in the second step of the cleaning processes, excepting S, which had a TF of 23% and 26% in Process A and B, respectively.

In relation with the activity concentrations of natural RNs after conducting the leaching test, the concentrations of ^210^Po and ^238^U were similar to those concentrations obtained for seawater, since the obtained concentrations were 0.0040 ± 0.0004 Bq/L for ^210^Po and 0.040 ± 0.002 Bq/L of ^238^U [30], with the potential radiological environmental impact being negligible.

### 3.4 Potential valorisation routes of the generated wastes

According to the discharges regulations, it is necessary to clean the acidic water inside the piles up to the established limits, so the most economical decontamination method must be found. In addition, the wastes generated during the cleaning treatment of PGL could be disposed in a landfill, but the new waste Law 7/2022, of April 8 [35] impulses the need for recycling and valorising the obtained wastes. This law focuses on prevention, preparation for reuse, recycling and other forms of recovery and considering the challenge of the circular economy.

If the valorisation of the generated waste is developed, a significant cost reduction will be achieved in relation to the traditional landfill disposal management. In addition, the environmental impact would be significantly reduced, making a significant contribution to the circular economy. In the current state of this research, it is not possible to quantify the economic implications of the global process (cleaning of the PGL and valorisation of generated waste), until the responsible company develops the engineering project by using the proposed process.In this section, the diagnoses for the valorisation of solid wastes are analysed, considering the comprehensive literature review.

#### 3.4.1 Solid from step 1

Considering the characterisation of these wastes from Step 1 and the literature consulted, two possibilities could be raised for the recovery of the fluorite. One of them is the study of the fluorite as additive in the manufacture of commercial Portland cement.

Many authors have studied the incorporation of calcium fluoride in the clinker for cement elaboration. Moreover, in the work of Lin et al. [36], mortar specimens with replacement of cement were studied (0%, 5%, 10%, 15%, 20%, 25%, and 30% based on weight percentage), with calcium fluoride sludge from industrial waste produced in the manufacturing of solar cells. The results indicated that partially replacing cement with calcium fluoride sludge improved the compressive strength, permeability, and pore-structure. In addition, 10% replacement of cement seemed to give superior mechanical properties and durability due to the denser microstructures formed.

Dominguez et al. [37] proved that when certain amount of CaF_2_ was added to the clinkerisation process, the mechanical properties were modified as the final amount of alite (Ca_3_SiO_5_) was greater. The highest compressive strength was achieved with 0.4 wt% of CaF_2_, increasing the compressive strength of the clinker paste from 30 MPa to almost 40 MPa. In addition, the evidence from this study suggested that CaF_2_ slightly affected CaCO_3_ decomposition, unlike belite (2CaO·SiO_2_), where CaF_2_ significantly affecting their corresponding temperatures. The calcium fluoride therefore acted as a carrier, decreasing the viscosity and surface tension of the oxide melt because it reduced the temperature at which the oxide was formed. Consequently, the alite was formed at lower temperature.

Proportions of 0.25%, 0.50%, and 1% of CaF_2_ and of 1%, 2%, and 3% of MgO were added to a mixture composed by limestone, sand and loam to improve the raw mix burning as these materials decrease the formation temperature of clinker minerals by increasing calcination, solid-state reaction, melt and alite formation rate [38]. The study concluded that the amount of CaO free decreased with increasing CaF_2_ content. The reason for the CaO free reduction was the increase in the speed of the solid-state reaction due to both the easy diffusion effect of fluorine and the acceleration of the necessary reactions for clinker phase formation due to the low viscosity of the molten fluorine.

Dahhou et al. [39] presented a new method to synthesise belite clinkers at lower temperatures by incorporating natural fluorite (CaF_2_) into a mixture of limestone and alumina sludge originated from water purification plants. The results indicated that the synthesised belite cement possessed mechanical strength like that of ordinary belite cement. In addition, the introduction of CaF_2_ into the clinkers not only lowered their preparation temperatures but also contributed to the formation of crystalline phases which eventually improved the hydraulic properties of the cement.

Additionally, the second possibility for the waste obtained in Step 1 was the manufacture of ceramic tiles from mixtures of a commercial red stoneware mixture (RSM) with different concentrations of waste.

Li_2_O–Al_2_O_3_–SiO_2_ (LAS) based glass-ceramics are widely studied because of their low coefficient of thermal expansion and adjustable crystallisation phase, thus enhancing both the reduction of thermal stress and the extension of service life. Feng et al. [40] studied the effects of CaF_2_ on the LAS based glass-ceramics and glass-ceramic/diamond composites. The addition of CaF_2_ decreased the temperature of refractoriness because the fluorine entered into a glass network and replaced non-bridge oxygen ions, thus weakening the glass network and relaxing the glass structure. The high temperature fluidity of glass binder was inversely proportional to the value of refractoriness. The study showed the benefits of the addition of calcium fluoride for the glass crystallisation and mechanical properties. Moreover, when the content of CaF_2_ reached 7 wt%, the nucleation temperature of the glass-ceramic was reduced to 9 °C, and the bending strength and the Rockwell hardness of the glass-ceramic/diamond composites were improved by 28% and 29%, respectively.

Furthermore, Wang et al. [41] showed ceramic composites based on the addition of nanosized CaF_2_ solid lubricants produced by vacuum hot pressing. Compared with the ceramic composite without CaF_2_, the results concluded that the mechanical properties of ceramics were improved, the flexural strength, hardness and fracture toughness reached a higher maximum, and the main cutting force and temperature were reduced by 13.2% and 26.9%, respectively.

#### 3.4.2 Solid from step 2

Water soluble fertilisers are the main P source used worldwide. They play an essential role in agriculture because they enable fast increase in P available in soil solution and in P bioavailability [42]. The phosphate mineral, mainly of the apatite group, is in the nature mixed with many impurities, such as clay, silica, calcite, dolomite, organic matter, and various other inorganic compounds. These impurities adversely affect the manufacture of phosphoric acid, so they must be reduced to the lowest level [43].

In the case of the second solid waste obtained in the neutralisation process, mainly composed by hydroxyapatite (Ca_5_(PO_4_)_3_OH), the calcium phosphate was already cleaned because the neutralisation process was applied and the most relevant contaminants were eliminated or drastically reduced as discussed in the previous section. Table 5 shows a comparison between the compositions of the current phosphate rocks used to produce phosphoric acid in different parts of the world. The obtained waste of this study (SA2 and SB2) had a similar component in raw material compared with the commercial phosphorite used from Morocco and Florida to produce phosphoric acid. Potential contaminants such as RNs and fluorine were significantly reduced. The HA generated in this study was approximately half the concentration of ^238^U, 30 times less than ^226^Ra, and 150 times less than ^232^Th. As for fluorine, which is considered as a residue in the manufacture process of phosphoric acid, the reduction was 2 orders of magnitude.

**Table 5:** Comparison of the chemical composition (%), activity concentation (Bq/kg) and mineralogy of phosphate rocks from different deposits around the world [44] with residues from Step 2 (SA2, SB2). N.D.: not detected.

	Florida (Phosphorite)	Morocco (Phosphorite)	SA2, SB2
**Chemical composition (%)**			
**SiO**_**2**_	11	2.1	2.4
**Al**_**2**_**O**_**3**_	1.8	0.6	0.02
**Fe**_**2**_**O**_**3**_	0.8	0.23	0.06
**MgO**	0.3	0.4	2.3
**CaO**	44	52	43
**P**_**2**_**O**_**5**_	30	33	30
**CO**_**2**_	N.D.	5.1	N.D.
**F**	3.2	4.0	0.01
**Activity concentration (Bq/kg)**			
^**238**^**U**	1500–1900	1500–1700	750
^**226**^**Ra**	1800	1500–1700	< 50
^**232**^**Th**	16–59	10–200	< 0.4
**Major minerals**	Hydroxyapatite	Carbonate fluorapatite	Hydroxyapatite

Further studies should evaluate both the use of this waste as a direct fertiliser and the obtaining of phosphoric acid through digestion with sulfuric acid, since the hydroxyapatite had no potential contaminants. The environmental advantages would be a lower use of raw material and a lower environmental impact of the final residue.

In both cases, waste must be valorised according to the Royal Decree 506/2013 of June 28, 2013 on fertiliser products [45]. This decree contains seven annexes detailing the technical specifications and other requirements that such products must meet to be used in Spanish agriculture and gardening.

## 4 Conclusions

This study deeply characterised the wastes generated in the optimised cleaning process of phosphogypsum leachates. There were two steps based on a calcium carbonate/hydroxide neutralisation process. Moreover, the diagnosis of the main potential applications of these wastes was analysed. The main results and conclusions were as follows.

1. The phosphogypsum leachate is a naturally occurring radioactive material from industry, so high radionuclide removal efficiency was achieved, thus reducing an effluent with concentrations by 3 orders of magnitude.
2. After the cleaning process, all the potential contaminants (Al, As, Cd, Cr, Cu, Fe, Mn, Ni, P, Pb, Se, Sn, Ti, Zn, F^−^, nitrite, and phosphate) were reduced, and the final effluent met the emission limits set by the EU. Additionally, the pH was reduced to 8–9 by applying the carbonation process, thus obtaining CaCO_3_.
3. The leachate test indicated that solids from the first step (SA1 and SA2) and SB1 could be catalogued as hazardous wastes. However, SB2 couldn't be disposed on landfill because its concentration of As in the leachate exceeded the limit of the standard.
4. According to the characterisation of waste and the consulted bibliography, two main routes of valorisation could be of interest:
a. Solid 1: this waste was composed by 37–55% of fluorite and could be used as an additive in the manufacture of construction materials, thus increasing the mechanical properties of new materials.
b. Solid 2: this waste was composed by around 80% of hydroxyapatite and could be used as raw material to obtain phosphoric acid. In addition, its concentrations of potential contaminants were lower than those of the phosphate rock, thus producing a PG residue with lower environmental impact.

## Data availability statement

The authors declare that the data supporting are not available in any repository, and they will be provided under request.

## CRediT authorship contribution statement

**F.J. Soto-Cruz:** Writing – review & editing, Writing – original draft, Visualization, Validation, Methodology, Investigation. **S.M. Pérez-Moreno:** Writing – review & editing, Writing – original draft, Validation, Supervision, Methodology, Investigation, Conceptualization. **E. Ceccotti:** Writing – original draft, Validation, Methodology, Investigation. **A. Barba-Lobo:** Writing – review & editing, Writing – original draft, Validation, Supervision, Data curation. **J.P. Bolívar:** Writing – review & editing, Supervision, Resources, Project administration, Funding acquisition, Conceptualization. **M. Casas-Ruiz:** Supervision, Resources, Project administration, Funding acquisition. **M.J. Gázquez:** Writing – review & editing, Supervision, Resources, Project administration, Funding acquisition, Conceptualization.

## Declaration of competing interest

The authors declare that they have no known competing financial interests or personal relationships that could have appeared to influence the work reported in this paper.
