# Supplementary material for: Valorisation diagnosis of waste from the decontamination of phosphogypsum leachates through a combined calcium carbonate/hydroxide process
Source: Heliyon. 2024 May 4;10(9):e30610. doi: 10.1016/j.heliyon.2024.e30610 (PMC11101815; doi:10.1016/j.heliyon.2024.e30610)
Supplement: Multimedia component 1 [file mmc1.docx]

# Title: Valorisation diagnosis of waste from the decontamination of phosphogypsum leachates through a combined calcium carbonate/hydroxide process

*F.J. Soto-Cruz^1*^, S. M. Pérez-Moreno^2^, A. Barba-Lobo^2^, E. Ceccotti^2^, M. Casas-Ruiz^1^, J. P. Bolívar^2^, M. J. Gázquez^1^*

^1^ Department of Applied Physics, Marine Research Institute (INMAR), University of Cadiz, Campus de Excelencia Internacional del Mar (CEIMAR), Cádiz, Spain.

^2^ Research Centre of Natural Resources, Health and the Environment (RENSMA), University of Huelva, Campus de Excelencia Internacional del Mar (CEIMAR), Huelva, Spain.

*Corresponding author.

E-mail address: [franciscojavier.soto@uca.es](mailto:franciscojavier.soto@uca.es)


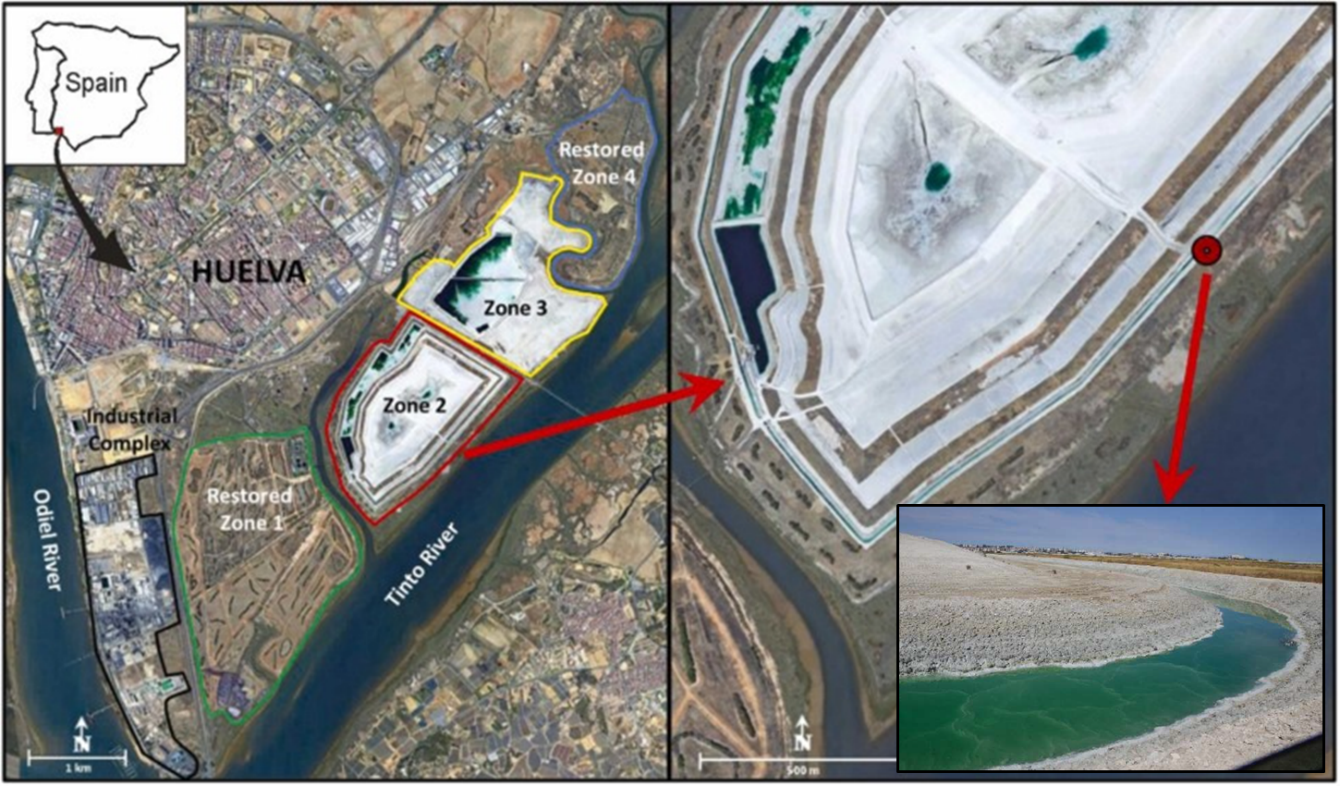
**Table S1**. Phosphogypsum leachate (PGL) composition and background value (seawater, SW) [10].

Figure S1. Location of the city, Industrial Complex and the PG stacks

| **Parameters** | **PGL** | **Background value (SW)** | **PGL/ SW** |
| --- | --- | --- | --- |
| **pH** | 1.7 | 7.8 | 0.20 |
| **EC (mS/cm)** | 30.2 | 61.0 | 0.50 |
| **Eh (mV)** | 656 | 460 | 1.40 |
| **Element (mg/L)** | | |  |
| **Al** | 6.5 | 0.130 | 50 |
| **As** | 27.4 | < 0.002 | 1.4·10^4^ |
| **Ca** | 1660 | 440 | 3.80 |
| **Cd** | 7.8 | < 0.002 | 3.9·10^3^ |
| **Cr** | 22.1 | < 0.002 | 1.1·10^4^ |
| **Cu** | 8.7 | 0.004 | 2.1·10^3^ |
| **Fe** | 129 | 0.008 | 1.6·10^4^ |
| **K** | 268 | 440 | 0.60 |
| **Mg** | 906 | 1440 | 0.60 |
| **Mn** | 12.4 | < 0.002 | 6.1·10^3^ |
| **Na** | 4920 | 11700 | 0.40 |
| **Ni** | 5.6 | < 0.002 | 2.8·10^3^ |
| **Pb** | 0.8 | 0.003 | 2.5·10^2^ |
| **Zn** | 65.9 | 0.050 | 1.3·10^3^ |
| **U** | 20.5 | 0.003 | 6.4·10^3^ |
| **Anions (mg/L)**  **C (mg/L)**  **C (mg/L)** | | |  |
| **F**^-^ | 1960 | 111 | 18 |
| **Cl**^-^ | 5250 | 21700 | 0.20 |
| **Br**^-^ | 28 | 69 | 0.40 |
| **PO**_4_^-^ | 31300 | 791 | 40 |
| **SO**_4_^-^ | 4540 | 3288 | 1.40 |
| **Radionuclides (Bq/kg)** | | |  |
| **^210^Po** | 39 ± 1 | 0.0036 ± 0.0004 | 1·10^4^ |
| **^238^U** | 254 ± 12 | 0.042 ± 0.002 | 6·10^3^ |
| **^234^U** | 248 ± 12 | 0.041 ± 0.002 | 6· 10^3^ |
| **^230^Th** | NM | 0.0016 ± 0.0001 |  |
| **^232^Th(ICP)** | 0.04 ± 0.06 |  |  |
| **^226^Ra** | < 2 | 0.0018 ± 0.0001 | - |
| **^210^Pb** | 60 ± 3 | 0.0036 ± 0.0004 | 2·10^4^ |

Table S2. Physicochemical data, elemental composition (mg/L) and anions (mg/L) of phosphogypsum leachate (PGL) and liquid fraction measured by ICP-OES and ICP-MS

|  |  | **Liquid fraction** | | | |
| --- | --- | --- | --- | --- | --- |
| **Parameters** | **PGL** | **Process A** | | **Process B** | |
|  |  | **LA1** | **LA2** | **LB1** | **LB2** |
| **pH** | 1.7 | 3.5 | 12 | 35 | 12 |
| **EC (mS/cm)** | 30.2 | 24.3 | 19.1 | 23.8 | 18.7 |
| **Eh (mV)** | 656 | 557 | 163 | 553 | 177 |
| **Elements (mg/L)** | | | | | |
| **Al** | 6.5 | 1.0 | 0.2 | 0.9 | 0.3 |
| **As** | 27.4 | 26.5 | 0.004 | 26.1 | 0.005 |
| **Ca** | 1660 | 4700 | 213 | 3900 | 180 |
| **Cd** | 7.8 | 7.3 | 0.002 | 7.0 | < 0.001 |
| **Cl** | 5250 | 5121 | 5770 | 5366 | 5752 |
| **Cr** | 22.1 | 3.6 | 0.03 | 1.8 | 0.03 |
| **Cu** | 8.7 | 5.3 | 0.07 | 4.6 | 0.085 |
| **F** | 1958 | 55.1 | 0.9 | 38.9 | 0.7 |
| **Fe** | 129 | 0.7 | < 0.1 | 0.3 | 0.1 |
| **K** | 268 | 252 | 227 | 251 | 204 |
| **Mg** | 906 | 875 | 0.7 | 886 | < 0.1 |
| **Mn** | 12.4 | 12.5 | 0.002 | 12.7 | 0.001 |
| **Na** | 4920 | 4881 | 4030 | 4847 | 4020 |
| **Ni** | 5.6 | 5.4 | 0.19 | 5.0 | 0.19 |
| **P** | 10900 | 9060 | 8.7 | 8602 | 5.7 |
| **Pb** | 0.8 | 0.005 | < 0.001 | 0.004 | < 0.001 |
| **S** | 1510 | 1440 | 236 | 1090 | 184 |
| **Se** | 0.08 | 0.06 | 0.04 | 0.05 | 0.04 |
| **Si** | 512 | 463 | 4.1 | 445 | 4.3 |
| **Th** | 0.04 | 0.01 | 0.0022 | < 0.0025 | < 0.001 |
| **U** | 20.5 | 3.6 | < 0.001 | 0.9 | < 0.001 |
| **Zn** | 65.9 | 45.6 | 0.009 | 38.6 | 0.005 |
| **Anions (mg/L)** | | | | | |
| **F**^-^ | 1958 | 55.3 | 0.9 | 38.8 | 0.7 |
| **Cl**^-^ | 5250 | 5122 | 5770 | 5367 | 5752 |
| **NO_2_^-^** | < 2 | < 1.4 | < 4 | < 1 | < 1 |
| **Br**^-^ | 28.1 | 23.1 | 18.7 | 24.8 | 18.6 |
| **NO_3_^-^** | 24.4 | 23.6 | 53.3 | 23.2 | 63.4 |
| **PO**_4_^-^ | 31334 | 27365 | 26.7 | 25901 | 17.5 |
| **SO**_4_^-^ | 4538 | 4082 | 645 | 3241 | 488 |

|  | **Solid fraction** | | | |
| --- | --- | --- | --- | --- |
| Parameters | Process A | | **Process B** | |
|  | SA1 | SA2 | **SB1** | **SB2** |
| **Major Elements (mg/kg)** | | | | |
| Ca | 365000 | 305000 | 357000 | 305000 |
| Cl | 1500 | 10300 | 400 | 7000 |
| F | 188000 | 0 | 130600 | 0 |
| Fe | 16000 | 400 | 13000 | 400 |
| K | 2000 | 1000 | 1000 | 2000 |
| Mg | 5000 | 14000 | 3000 | 14000 |
| Na | 7000 | 24000 | 3000 | 23000 |
| P | 72000 | 124000 | 67000 | 136000 |
| S | 5000 | 23000 | 32000 | 18000 |
| Si | 200 | 11000 | 200 | 11000 |
| **Minor Elements (mg/kg)** | | | | |
| Al | 1000 | 100 | 800 | 100 |
| As | 133 | 475 | 95.3 | 517.5 |
| Cd | 71.7 | 141.5 | 65.5 | 154 |
| Cr | 2115 | 58.5 | 1680 | 29 |
| Cu | 457.5 | 72.6 | 385.5 | 72.1 |
| Mn | 118 | 222.5 | 119 | 232 |
| Ni | 5.9 | 83.3 | 2.9 | 97 |
| Pb | 44.1 | 0.5 | 66.4 | 0.5 |
| Se | 0.65 | 0.5 | 0.7 | 0.4 |
| Sr | 1000 | 509 | 1000 | 553 |
| Th | 0.1 | 0.1 | 0.1 | 0.1 |
| U | 1820 | 49.8 | 1500 | 13.5 |
| Zn | 2545 | 677 | 2340 | 640 |

Table S3. Elemental composition (mg/L) of the solid fraction (SA1, SA2, SB1, SB2) measured by ICP-OES and ICP-MS


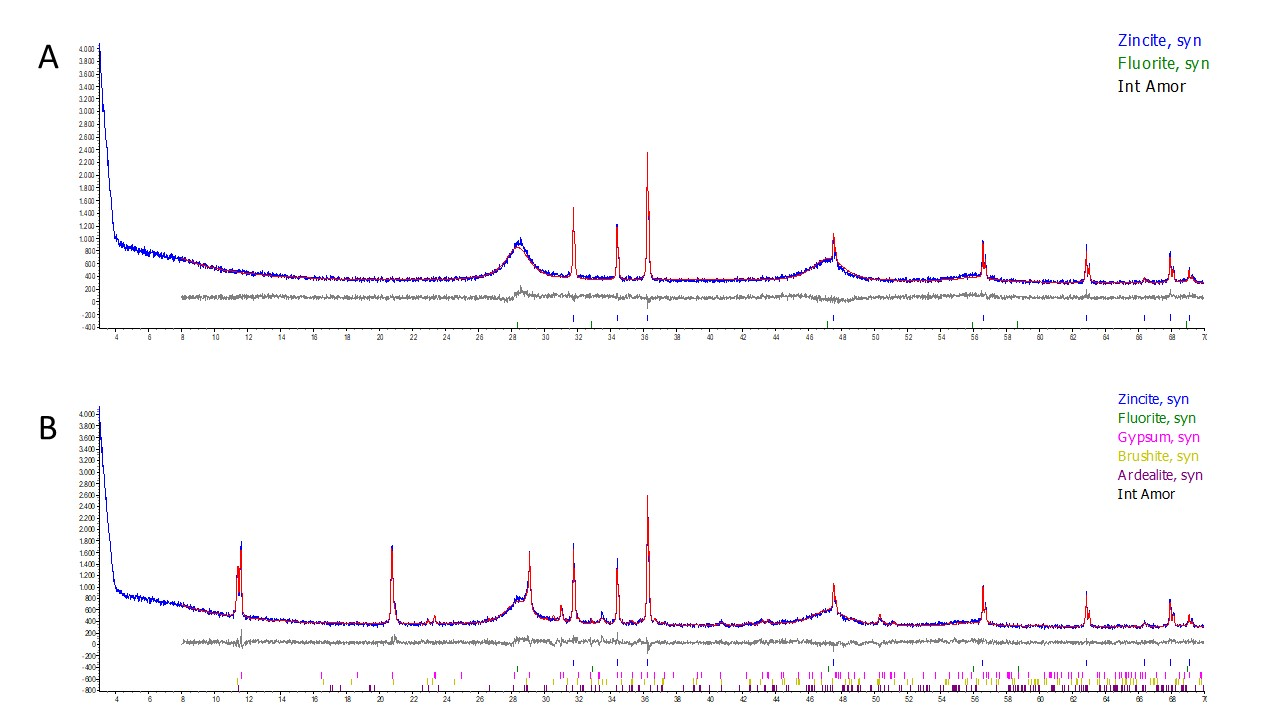
Figure S2. X-ray diffraction (XRD) patterns of solids from step 1: A: process A (SA1). B: process B (SB1)

Figure S3: X-ray diffraction (XRD) patterns of solids from step 2: A: process A (SA2). B: Process B (SB2)


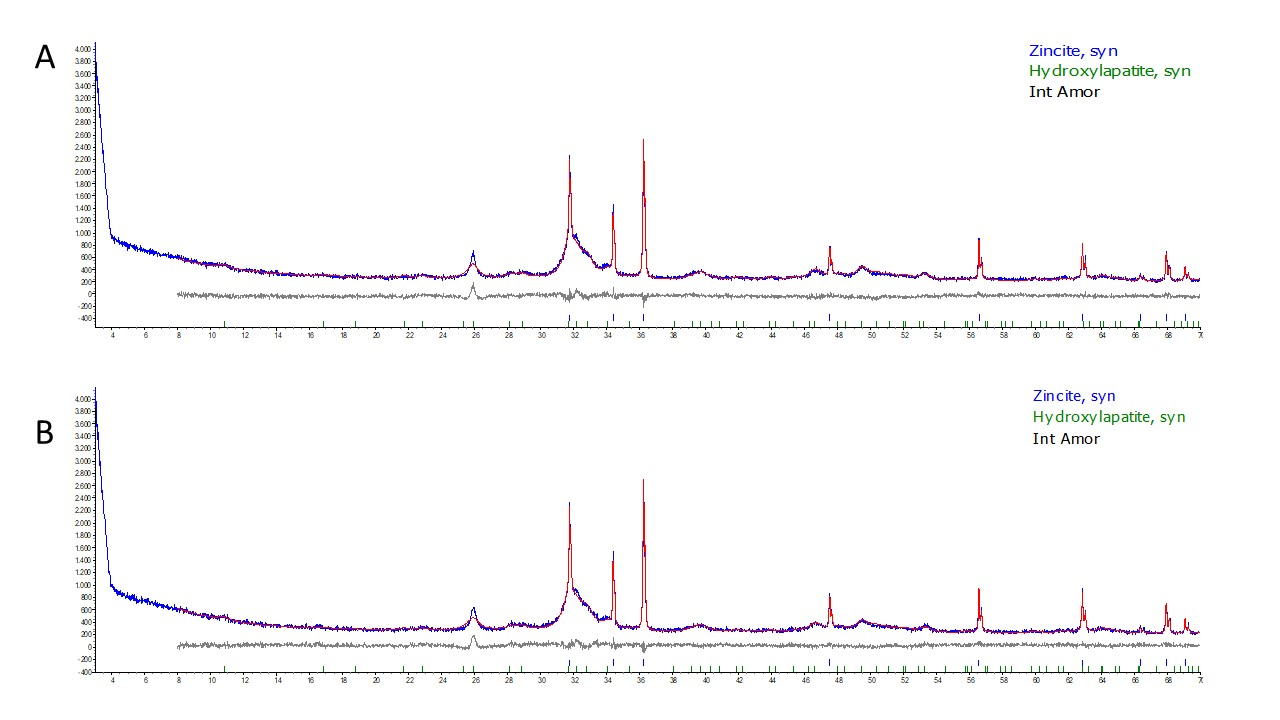


Figure S4. Calcite precipitated after bubbling the final liquid with air.


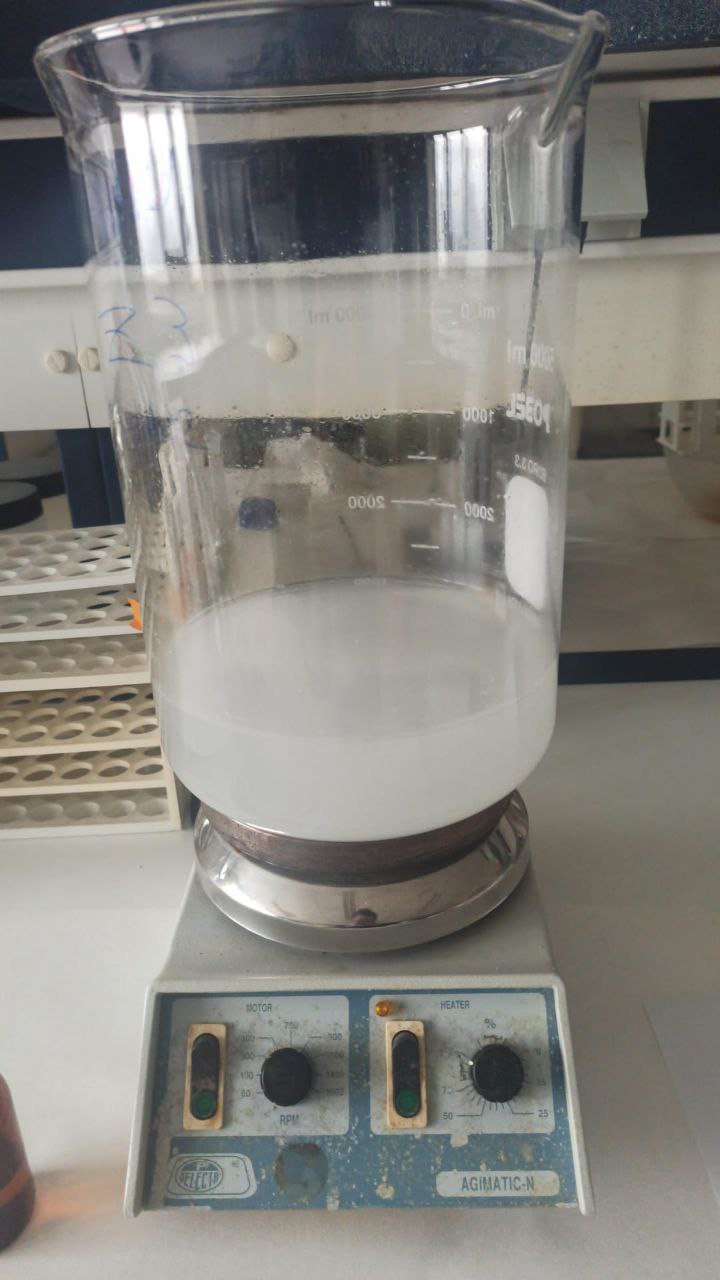


Figure S5. X-ray diffraction (XRD) patterns of Calcite from the reduction of pH. A: process A. B: Process B


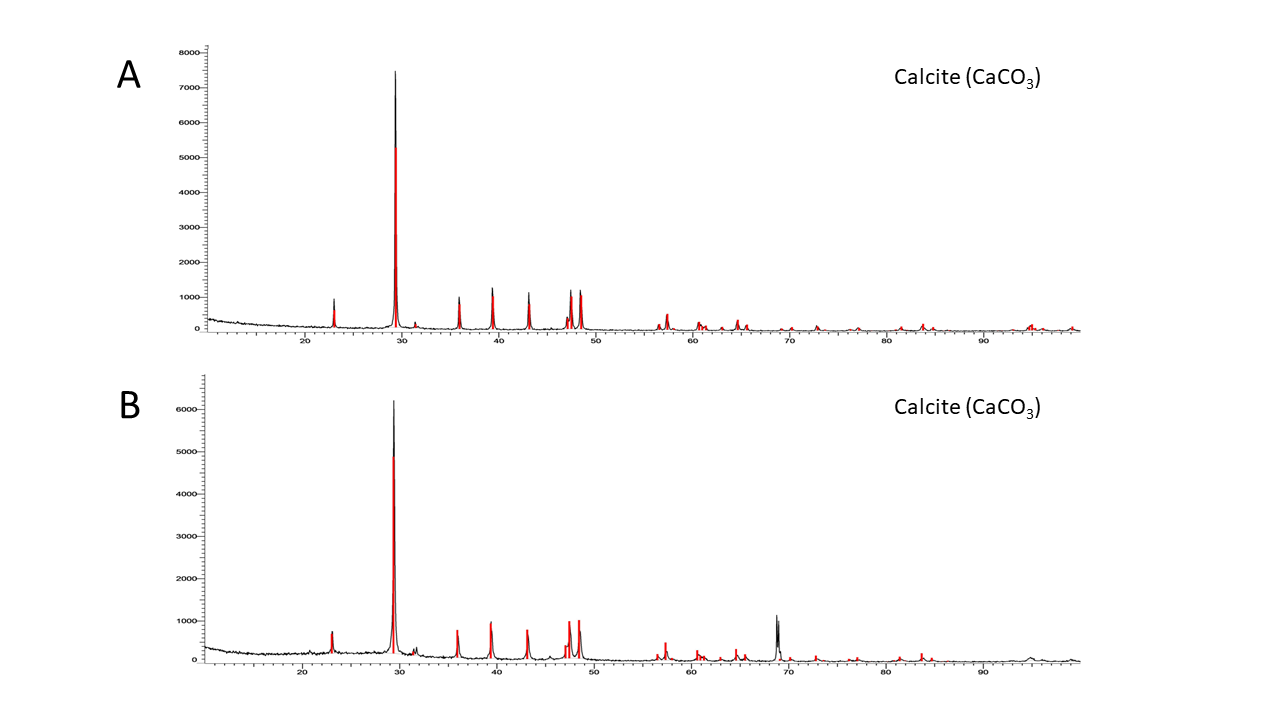


Table S4. Transfer factor of the residues generated in the decontamination process (%)

| **Element** | **SA1** | **SA2** | **LA2** | **SB1** | **SB2** | **LB2** |
| --- | --- | --- | --- | --- | --- | --- |
| **Al** | 55.7 | 43.0 | 1.3 | 60.4 | 37.9 | 1.7 |
| **As** | 3.5 | 96.5 | 0.0 | 3.5 | 96.5 | 0.0 |
| **Ca** | 13.3 | 85.8 | 0.9 | 18.8 | 80.5 | 0.7 |
| **Cd** | 6.2 | 93.8 | 0.0 | 7.8 | 92.2 | 0.0 |
| **Cl** | 0.2 | 10.2 | 89.6 | 0.1 | 7.6 | 92.3 |
| **Cr** | 82.3 | 17.6 | 0.1 | 91.9 | 8.0 | 0.1 |
| **Cu** | 44.6 | 54.6 | 0.8 | 51.1 | 48.1 | 0.8 |
| **F** | 99.9 | 0.0 | 0.1 | 100.0 | 0.0 | 0.0 |
| **Fe** | 83.8 | 16.2 | 0.1 | 86.6 | 13.4 | 0.0 |
| **K** | 5.4 | 20.7 | 73.9 | 3.8 | 38.4 | 57.8 |
| **Mg** | 4.4 | 95.5 | 0.1 | 4.1 | 95.9 | 0.0 |
| **Mn** | 6.4 | 93.6 | 0.0 | 9.3 | 90.7 | 0.0 |
| **Na** | 1.4 | 3.6 | 95.0 | 0.7 | 27.7 | 71.5 |
| **Ni** | 0.9 | 95.7 | 3.4 | 0.6 | 96.6 | 2.8 |
| **P** | 7.0 | 92.9 | 0.1 | 8.9 | 91.0 | 0.1 |
| **Pb** | 91.7 | 8.0 | 0.3 | 96.2 | 3.6 | 0.1 |
| **S** | 2.4 | 84.1 | 13.5 | 23.5 | 66.5 | 10.0 |
| **Se** | 6.9 | 41.2 | 51.8 | 12.3 | 35.4 | 52.2 |
| **Si** | 0.2 | 99.2 | 0.6 | 0.4 | 99.1 | 0.6 |
| **Sr** | 19.7 | 77.2 | 3.1 | 26.2 | 72.8 | 1.1 |
| **Th** | 8.8 | 67.8 | 23.4 | 14.8 | 74.3 | 11.0 |
| **U** | 82.6 | 17.4 | 0.0 | 95.7 | 4.3 | 0.0 |
| **Zn** | 32.7 | 67.2 | 0.0 | 42.1 | 57.9 | 0.0 |

Table S5. Composition of reagents used in sequential optimization of the neutralization process, measured by ICP-MS (mg/kg and µg/kg)

|  | **Element** | **CaCO_3_** | **Ca(OH)_2_** |
| --- | --- | --- | --- |
| mg/kg | Al | 2.8 | 3.1 |
|  | Ca | 3590 | 4650 |
|  | Fe | < 0.015 | < 0.015 |
|  | K | 7.3 | 4.8 |
|  | Mg | 17.0 | 44.8 |
|  | P | 1.3 | 0.8 |
|  | S | 2.1 | 1.1 |
|  | Si | < 0.015 | < 0.015 |
|  | Na | 15.7 | 9.9 |
| µg/kg | Sc | 3.9 | 3.9 |
|  | Ti | 129.6 | 297.4 |
|  | V | 98.7 | 123.1 |
|  | Cr | 318.0 | 380.5 |
|  | Mn | 839.4 | 2020 |
|  | Co | 17.1 | 20.9 |
|  | Ni | 256.9 | 330.5 |
|  | Cu | 833.0 | 619.0 |
|  | Zn | 135.6 | 99.5 |
|  | As | 119.2 | 188.6 |
|  | Sr | 1780 | 2710 |
|  | Cd | 4.1 | 1.4 |
|  | Pb | 36.3 | 7.1 |
|  | Th | 0.4 | 0.5 |
|  | U | 8.7 | 2.0 |
